# Real-world treatment sequencing and effectiveness of second- and third-generation ALK tyrosine kinase inhibitors for *ALK*-positive advanced non-small cell lung cancer

**DOI:** 10.1016/j.lungcan.2024.107919

**Published:** 2024-08-03

**Authors:** Jessica R. Bauman, Geoffrey Liu, Isabel Preeshagul, Stephen V. Liu, Barbara Melosky, Devin Abrahami, Benjamin Li, Despina Thomaidou, Kirsten Duncan, Stan Krulewicz, Martin Rupp, Jessica J. Lin

**Affiliations:** a Department of Hematology and Oncology, Fox Chase Cancer Center, 333 Cottman Avenue, Philadelphia, PA 19111, United States; b Medical Oncology and Hematology, Princess Margaret Cancer, 200 Elizabeth Street, Toronto, ON M5G 0A3, Canada; c Thoracic Oncology, Memorial Sloan Kettering Cancer Center, 225 Summit Avenue, Montvale, NJ 07645, United States; d Thoracic Oncology, Georgetown University, 37th and O Streets NW, Washington, DC 20057, United States; e Department of Medicine, BC Cancer - Vancouver, 2775 Laurel Street, Vancouver, BC V5Z 1M9, Canada; f HTA Value and Evidence, Oncology, Pfizer, 66 Hudson Boulevard, New York, NY 10001, United States; g Biostatistics, Pfizer, 66 Hudson Boulevard, New York, NY 10001, United States; h Global Medical Affairs, Pfizer, 243 Mesogeion Avenue, Neo Psychiko, Athens 154 51, Greece; i US Medical Affairs, Pfizer, 66 Hudson Boulevard, New York, NY 10001, United States; j Medical Affairs Oncology, Pfizer Canada, 17300 Trans-Canada Highway Kirkland, Québec, H9J 2M5, Canada; k Center for Thoracic Cancers, Department of Medicine, Cancer Center, Massachusetts General Hospital, 55 Fruit Street, Boston, MA 02114, United States

**Keywords:** Advanced NSCLC, ALK TKIs, Real-world data, Sequencing, Targeted therapy, Treatment effectiveness

## Abstract

**Introduction:**

With multiple targeted therapies approved for anaplastic lymphoma kinase (*ALK*)-positive metastatic non-small cell lung cancer (NSCLC), it is increasingly important to understand outcomes with various sequences of next-generation ALK tyrosine kinase inhibitors (TKIs). We describe contemporary sequencing patterns and treatment effectiveness of first-line (1L) and second-line (2L) treatments in patients who received second-generation ALK TKIs in the 1L treatment of *ALK*-positive NSCLC in the United States.

**Methods:**

A cohort of adults with *ALK*-positive advanced NSCLC who initiated treatment with 1L alectinib or brigatinib between June 2017 and April 2021 in the Flatiron Health electronic health record–derived de-identified database were followed through April 2023. Time to treatment discontinuation (TTD) in 1L and 2L, TTD on 1L plus 2L sequential therapy (TTD2), and total time on sequential ALK TKI therapy (including beyond 2L) were evaluated.

**Results:**

Patients (N=273) were followed up for a median duration of 28.9 months. Among patients who discontinued 1L therapy, 22% died after 1L discontinuation (median time from discontinuation to death, 4.0 months) without receiving 2L therapy. Median (95% confidence interval [CI]) TTD was 21.9 (15.2–25.8) and 7.3 (5.3–10.2) months in 1L and 2L, respectively. Median (95% CI) TTD2 was 29.4 (25.1–36.1) months and total time on sequential ALK TKI treatment was 28.0 (23.6–32.9) months.

**Conclusions:**

In this large real-world study, TTD2 and the total time on sequential ALK TKIs was approximately 2.5 years. The high attrition rate from 1L to 2L and the longest clinical benefit observed with 1L therapy support using the drug with the longest 1L effectiveness up front in patients with *ALK*-positive advanced NSCLC.

## 1. Introduction

Anaplastic lymphoma kinase (*ALK*) gene rearrangements occur in 3% to 5% of patients with non-small cell lung cancer (NSCLC) and confer sensitivity to treatment with ALK tyrosine kinase inhibitors (TKIs) [1,2]. Current guidelines recommend alectinib (second-generation), brigatinib (second-generation), or lorlatinib (third-generation) ALK TKIs as the preferred first-line (1L) treatment for *ALK*-positive advanced or metastatic NSCLC [3-5]. In phase 3 randomized clinical trials of second-generation ALK TKIs (alectinib, brigatinib) in patients with treatment-naive *ALK*-positive NSCLC, median progression-free survival (PFS) was 25.7 months with alectinib and 24.0 months with brigatinib by independent review committee/blinded independent central review (BICR), or 34.8 months and 30.8 months by investigator assessment, respectively [6-8]. In the phase 3 CROWN trial of third-generation ALK TKI (lorlatinib) in patients with previously untreated *ALK*-positive advanced NSCLC, after a median follow-up of 60.2 months, median PFS by investigator assessment has not yet been reached with lorlatinib, with a 5-year PFS rate of 60% [9].

Among patients with *ALK*-positive metastatic NSCLC who are eligible to receive second-line (2L) treatment, the recommended treatments following progression on second-generation ALK TKIs are lorlatinib or chemotherapy [3,10]. In a phase 2 trial in patients who progressed following treatment with second-generation ALK TKIs, lorlatinib showed an overall response rate of 39.6% (95% confidence interval [CI], 31.4%-48.2%) and median PFS of 6.6 months (95% CI, 5.4–7.4) [11]. Indeed, the initial accelerated Food and Drug Administration approval of lorlatinib in 2018 was granted for patients with *ALK*-positive metastatic NSCLC who had received prior ALK TKI(s). With the option of using lorlatinib as later-line therapy, as well as differences in the adverse event profile, practice patterns of initial therapy for metastatic *ALK*-positive NSCLC have varied.

In studies of 1L use of second-generation ALK TKIs, a substantial proportion of patients (25%-41%) do not receive a 2L treatment at disease progression, mainly due to rapid clinical deterioration [7,8,12,13]. This raises the question of whether sequencing of therapies (e.g., second-generation ALK TKI followed by lorlatinib or chemotherapy) versus the utilization of the most efficacious therapy up front represents the optimal therapeutic approach to improve patients’ outcomes. Contemporary sequencing patterns, treatment effectiveness, and total time on sequential ALK TKI treatment for patients initiating 1L next-generation ALK TKI (i.e., second- or third-generation) have not yet been described in the real-world setting, and clinical trials report limited data on sequential outcomes [7-9]. Given the rarity of patients with metastatic NSCLC harboring *ALK* rearrangements, clinical trials may not be able to address such questions. Well-designed real-world studies can provide clinically useful data to supplement clinical trial information. Thus, the objective of this large, retrospective, real-world study is to describe sequencing patterns and treatment effectiveness of 1L and 2L treatments in patients with *ALK*-positive advanced NSCLC who initiated 1L alectinib or brigatinib treatment in routine clinical practice in the United States (US).

## 2. Methods

### 2.1. Data source

This study was conducted using the nationwide Flatiron Health electronic health record (EHR)–derived de-identified database. The Flatiron Health EHR database is a longitudinal database derived from EHR data from over 280 cancer clinics (approximately 800 sites of care) within the US [14-16] and contains information from more than 3 million active patients with cancer from all 50 US states and Puerto Rico [14].

### 2.2. Study population

A cohort of patients with *ALK*-positive advanced NSCLC who initiated treatment with 1L alectinib or brigatinib monotherapy from June 1, 2017, to April 30, 2021, with potential follow-up through April 30, 2023 (data cutoff), was assembled using the Flatiron EHR. The study start date corresponded to the release of the ALEX clinical trial results of alectinib in June 2017 and the incorporation of alectinib into treatment guidelines [6]. The date of the initiation of alectinib or brigatinib monotherapy treatment within the study period was considered the index date. At the index date, patients were required to be at least 18 years old, have a prior diagnosis of advanced NSCLC (stage IIIB or IV), and have a positive *ALK* gene rearrangement status. Patients who had received prior systemic treatments for metastatic disease, including treatment with any ALK TKIs, were excluded. Additionally, patients who received lorlatinib in 1L were not in scope of this study as lorlatinib was not approved in 1L until 2021; thus, these patients did not have substantial follow up to meaningfully contribute to the analysis. The cohort entry cutoff being in 2021 allowed up to 2 years of potential follow-up data for all patients at the data cutoff of 2023.

### 2.3. Clinical and demographic variables

Baseline covariates were assessed using records as close as possible to the index date (up to 12 months before the index date and 1 week after the index date). Baseline demographics included age, sex, race, smoking history, histology, practice type, Eastern Cooperative Oncology Group performance status (ECOG PS), ALK test type, and ALK sample type (blood, tissue, other). Patients were followed from their index date to the earliest date of treatment sequence discontinuation (i.e., end of 1L or 2L treatment), disenrollment, death, or end of the study period, whichever came first.

### 2.4. Exposure definition

Exposure was defined based on Flatiron’s treatment episode tables, in which the start of the episode corresponds to the first recorded date of a specific treatment as abstracted from a patient’s chart (i.e., date on which patients started the medication), and the end of the episode is abstracted from the chart as a treatment end date or is recorded as the day prior to initiation of subsequent therapy. For oral therapies, data were abstracted from unstructured patient chart records and corroborated with structured data to confirm that patients were actively receiving the drug. For intravenous medications (allowed in 2L and beyond), data were sourced from the administration date, expected start date, or medication ordered date. Exposure data were abstracted from both structured and unstructured data to maximize completeness. To determine patients who received an eligible 2L treatment (i.e., addition of another lung cancer treatment), a 60-day grace period was applied, starting from the end of the 1L treatment episode. If a patient received a subsequent therapy within the 60-day grace period, this was considered an eligible 2L therapy, and the patient was followed up until the end of their 2L treatment, death, or the end of study period. If there was no subsequent treatment within the grace period, the patient was censored at the end of the grace period. The 60-day grace period was selected as a reasonable time limit according to the scientific literature and clinical opinion during which patients would be eligible for a subsequent treatment, given that these patients were being treated for advanced NSCLC and were expected to receive continuous treatment [17]. The grace period was varied in sensitivity analyses to 30, 90, and 120 days. 60 days was selected as the primary grace period based on clinical practice and patient care standards, whereby patients are expected to receive overall continuous treatment, while also balancing the real-world limitations. For patients with recorded oncology treatments used for tumor types other than NSCLC, clinical opinion was sought to determine if the change or addition of systemic treatments met the eligibility criteria for 2L ALK TKI therapies for NSCLC on a case-by-case basis.

**Fig. 1.**
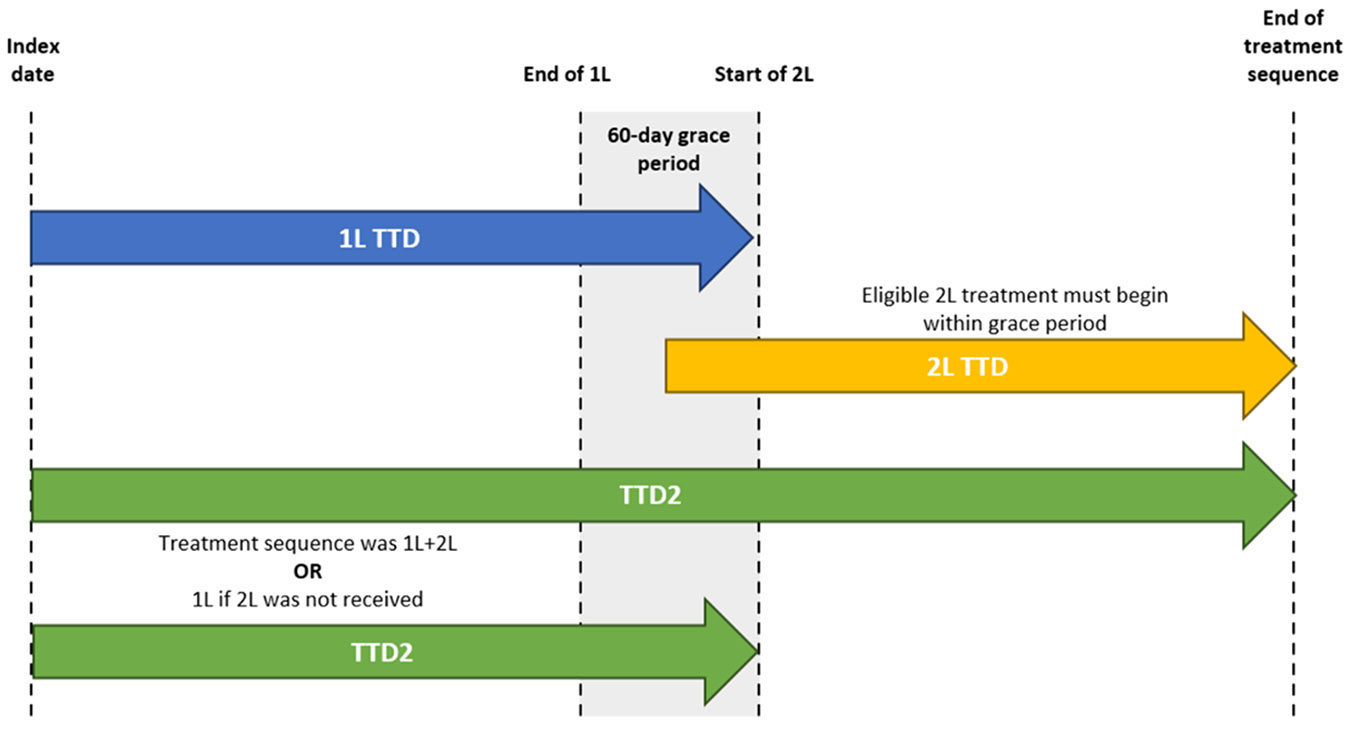
Diagram defining TTD and TTD2.^a^ 1L, first-line; 2L, second-line; TTD, time to treatment discontinuation; TTD2, time to treatment discontinuation sequence. ^a^ 1L TTD included the grace period up until the start of 2L treatment. If patients did not receive a 2L treatment within the grace period, they were censored at the end of the grace period. TTD was considered time from index until end of treatment episode (including the grace period), switch or add-on to new therapy, or death, whichever came first.

Treatment sequencing patterns were explored in all patients who initiated 1L therapy with alectinib or brigatinib. Treatment effectiveness was measured using time to treatment discontinuation (TTD), a pragmatic clinical endpoint for real-world studies often used in the literature that is closely associated with PFS [18]. TTD was defined as the time from the index to the end of the treatment episode (including the grace period for 1L), switch or add-on to new therapy, or death, whichever came first (Fig. 1). While this endpoint is closely associated with PFS, progression could not be assessed as a reason for discontinuation. In addition, time to treatment discontinuation sequence (TTD2) was measured from the index date to the end of the sequence treatment episode to quantify the effectiveness of the treatment sequence. The end of treatment episode was either the end of the duration of 1L plus subsequent 2L treatments or the end of 1L therapy (including grace period) if a patient did not receive a subsequent treatment. Finally, time on ALK TKI therapy was defined as the time from the index date to discontinuation of ALK therapy. Time on ALK TKI therapy was considered in any line of therapy (i.e., including beyond 2L), with the initiation of any non-ALK therapy considered as the end of the treatment episode. This definition was altered in sensitivity analyses to allow for short durations of chemotherapy (<8 weeks or 2 cycles) and combination ALK TKI therapies.

### 2.5. Statistical analysis

Patient characteristics were quantified at the index date using descriptive statistics as median (first quartile [Q1], third quartile [Q3]) for continuous variables and counts (percentages) for categorical variables. Demographics were also quantified for patients who discontinued 1L therapy within 12 months from the index date (i.e., early discontinuation) and for patients who discontinued 1L therapy without subsequent receipt of 2L therapy. An early discontinuation definition of ≤12 months was chosen as it was less than half of the median PFS for alectinib (ALEX trial) [6] and brigatinib (ALTA-1L) [7], representing a clinically relevant subgroup of patients with poor prognosis.

1L TTD, 2L TTD, and TTD2 were summarized descriptively. 1L TTD and TTD2 were stratified by ECOG PS (0–1, 2+, missing), practice type (academic, community, both), age (<65 years, 65+ years), and sex (male, female). 2L TTD was stratified by receipt of ALK TKI (including combination therapies) or non–ALK TKI therapy in the 2L. TTD2 was stratified according to type of medication received in the 2L (third-generation ALK TKI vs other). Time-to-event endpoints (TTD, TTD2) were also estimated using the Kaplan-Meier methodology. Patients who remained on treatment at the end of enrollment or at the end of the study period were censored from this analysis at the time of their last followup.

To explore the relationship between discontinuation of 1L therapy and death as the reason for not starting 2L therapy, the median time to death was investigated as the time from the end of 1L therapy to death, occurring at any time during the study period. Patients who did not die were censored if they received a 2L therapy at any time, were lost to follow-up, or were administratively censored at the end of the study period, whichever came first. All analyses were conducted using SAS software (SAS Institute Inc, Version 9.4).

## 3. Results

### 3.1. Baseline characteristics

**Fig. 2.**
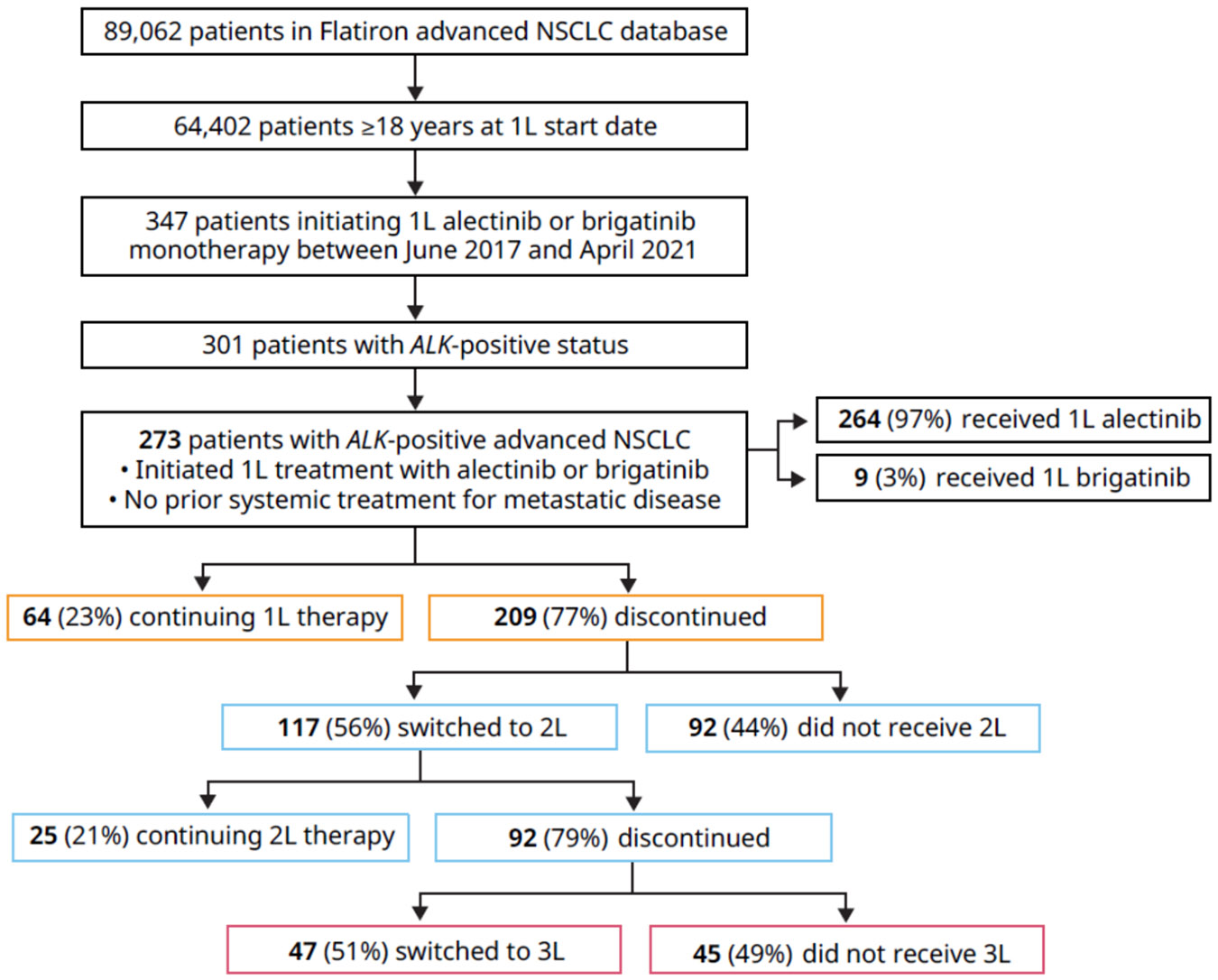
Patient flow chart. 1L, first-line; 2L, second-line; 3L, third-line; NSCLC, non-small cell lung cancer.

This study included 273 patients with *ALK*-positive advanced NSCLC who initiated 1L therapy with second-generation ALK TKIs from June 1, 2017, through April 30, 2021 (Fig. 2). Table 1 shows the baseline demographics and clinical characteristics of patients initiating 1L alectinib or brigatinib. The median (Q1, Q3) age was 64 (53, 73) years, 58% of the patients were female, 64% were White, 55% had no history of smoking, and 97% had non-squamous cell carcinoma. Most patients had their ALK testing by fluorescence in situ hybridization (53%) or next-generation sequencing (33%). The majority of ALK testing was performed on tissue (81%). Patient characteristics were stratified by patients who discontinued 1L therapy within 12 months (n = 96) or continued beyond 12 months (n = 177; Supplementary Table S1) and those who did not receive a 2L therapy (n = 92; Supplementary Table S2).

### 3.2. Sequencing patterns

**Fig. 3.**
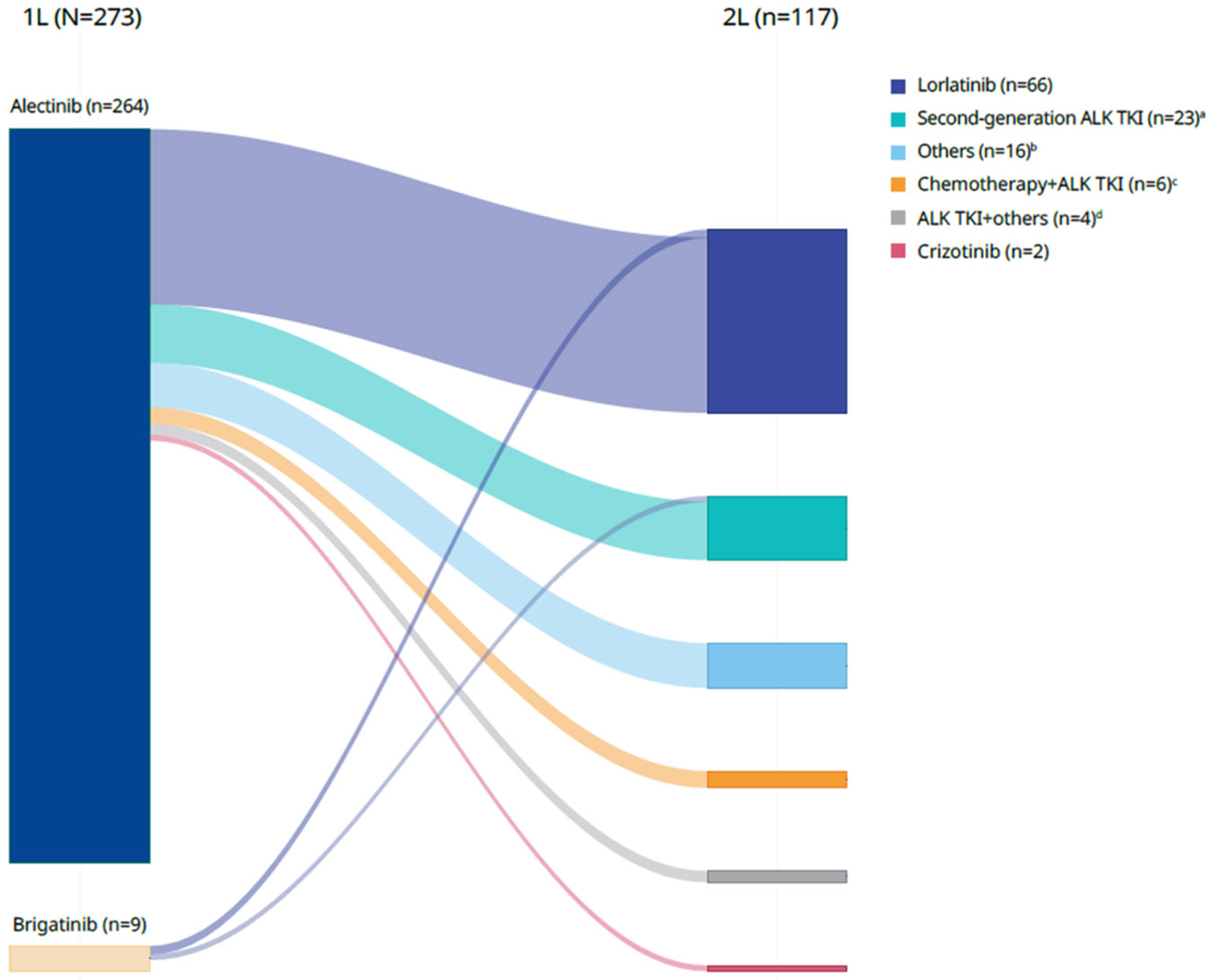
Sankey diagram illustrating treatment sequences of 1L to 2L therapy in patients with *ALK*-positive NSCLC. 1L, first-line; 2L, second-line; ALK, anaplastic lymphoma kinase; NSCLC, non-small cell lung cancer; TKI, tyrosine kinase inhibitor. ^a^ Second-generation ALK TKIs were brigatinib (n = 18), ceritinib (n = 3), and alectinib (n = 2); alectinib was 2L only in patients receiving 1L brigatinib. ^b^ Other treatments were carboplatin, pembrolizumab, and pemetrexed (n = 6); clinical study drug (n = 4); pembrolizumab (n = 3); carboplatin and paclitaxel (n = 2); and carboplatin and pemetrexed (n = 1). ^c^ Chemotherapy + ALK TKI were alectinib, carboplatin, and pemetrexed (n = 2); alectinib, carboplatin, and gemcitabine (n = 1); alectinib, carboplatin, and paclitaxel (n = 1); alectinib, cisplatin, and pemetrexed (n = 1); and lorlatinib, carboplatin, and pemetrexed (n = 1). ^d^ ALK TKI + others were alectinib and clinical study drug (n = 1), alectinib and pembrolizumab (n = 1), lorlatinib and clinical study drug (n = 1), and lorlatinib and bevacizumab (n = 1).

Of the study cohort, 264 patients (97%) received 1L alectinib and 9 (3%) received 1L brigatinib. Treatment patterns are presented in Fig. 3. Of the 117 patients who received 2L therapy, the majority (n = 101, 86%) received ALK TKI–based therapy in the 2L setting. Lorlatinib and brigatinib were the most commonly used 2L therapies, prescribed to 66 (56%) and 18 patients (15%), respectively. Fifteen patients (13%) received 2L chemotherapy, of whom 6 (40%) continued on an ALK TKI together with chemotherapy. The most frequently prescribed treatment sequence was 1L alectinib followed by 2L lorlatinib (Fig. 2). The median (Q1, Q3) duration of follow-up from 1L initiation was 28.9 (14.4, 45.8) months.

### 3.3. Outcomes of 1L therapy with second-generation ALK TKI

**Fig. 4.**
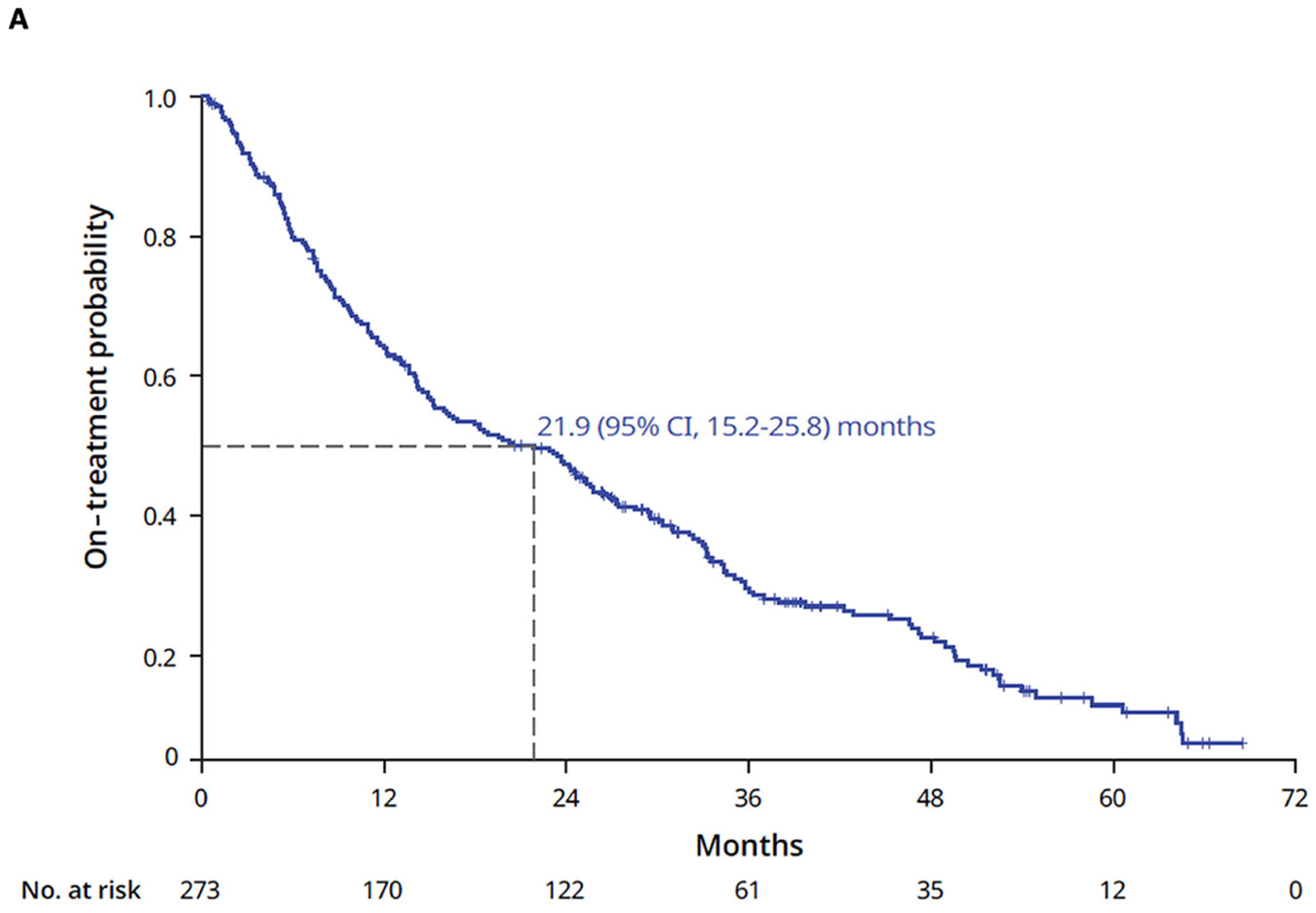

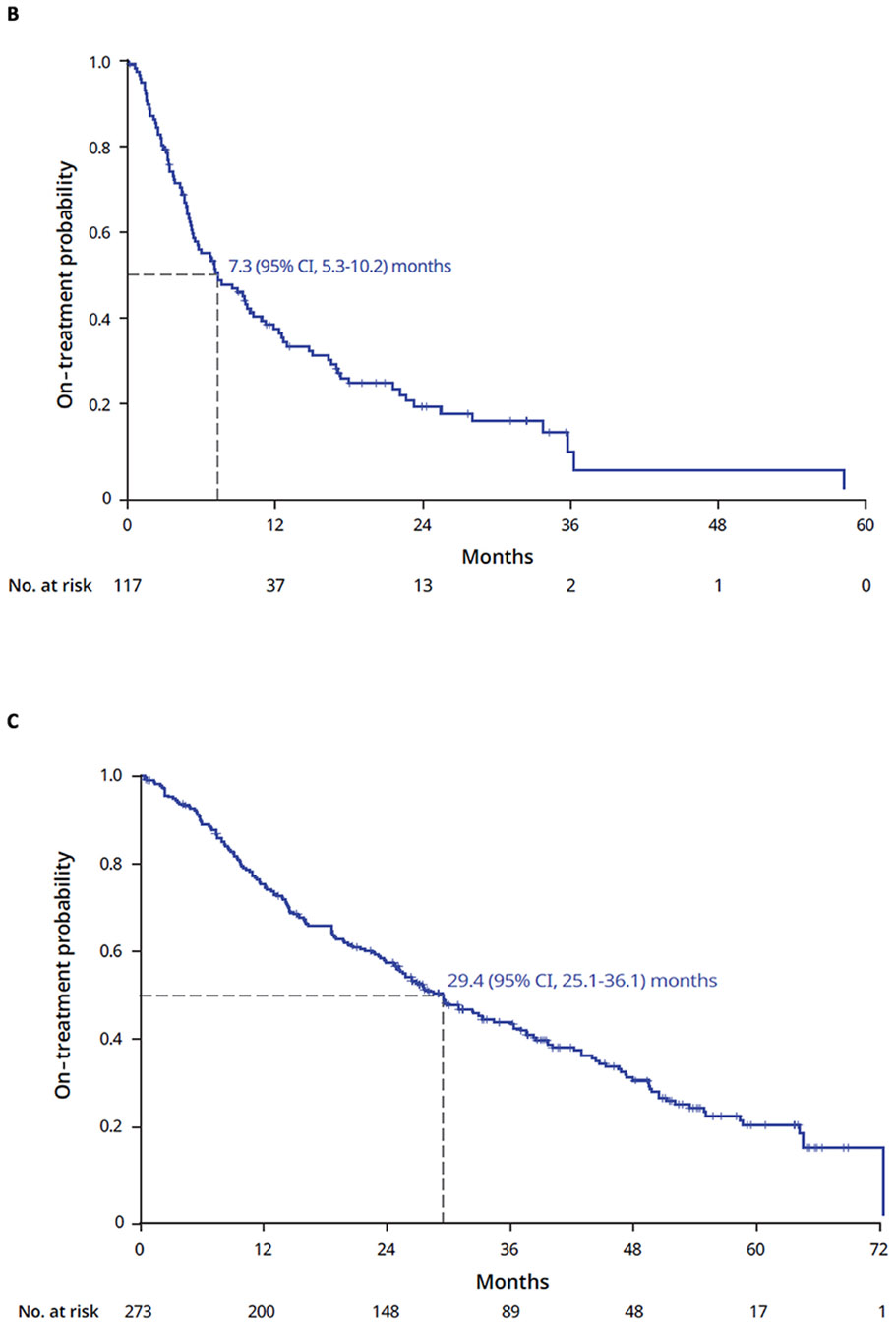

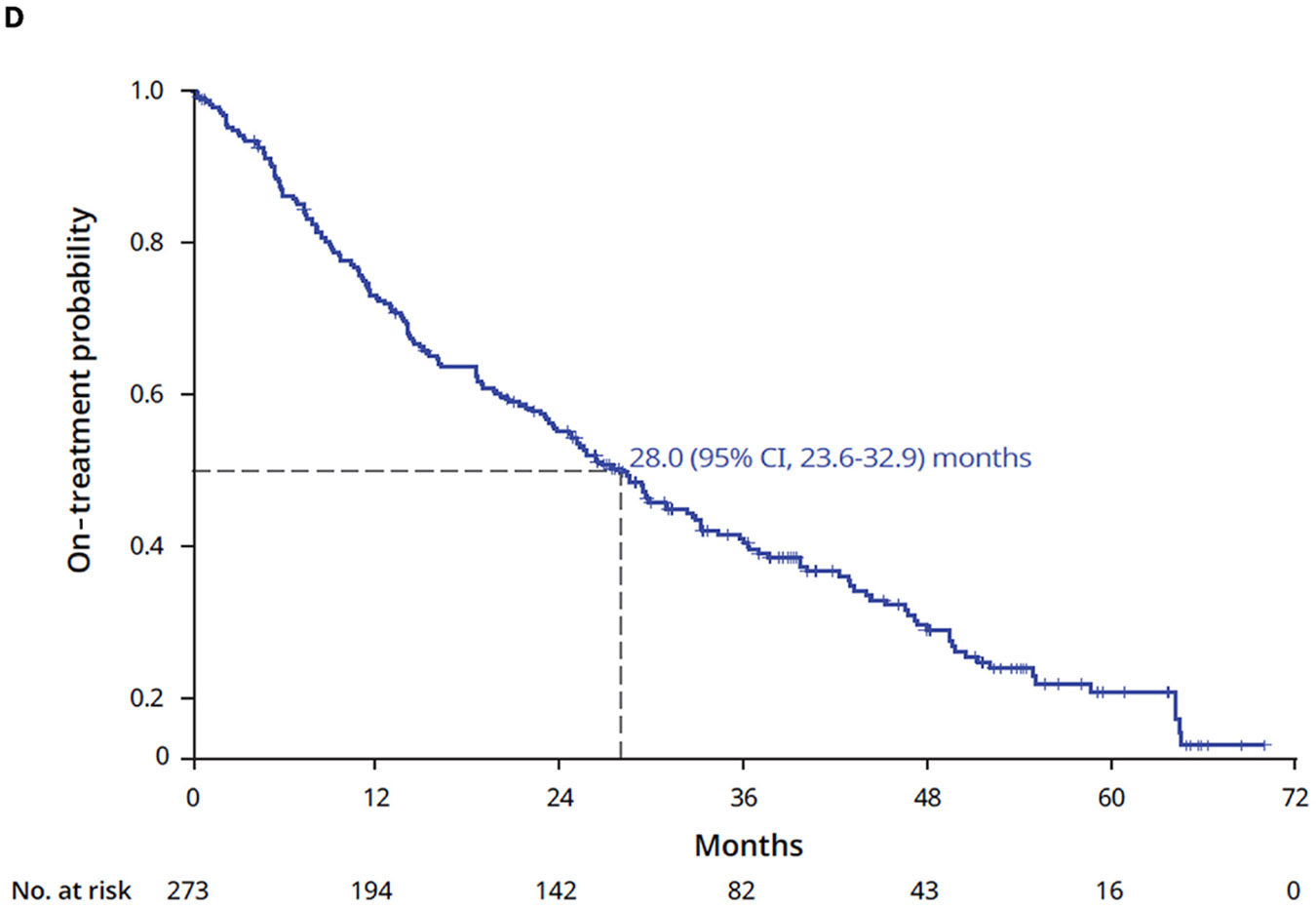
Kaplan-Meier analysis of TTD in patients with *ALK*-positive advanced NSCLC receiving 1L alectinib or brigatinib monotherapy. (A) 1L TTD, (B) 2L TTD, (C) TTD2, and (D) TTD of sequential ALK TKI treatment. 1L, first-line; 2L, second-line; ALK, anaplastic lymphoma kinase; CI, confidence interval; NSCLC, non-small cell lung cancer; TKI, tyrosine kinase inhibitor; TTD, time to treatment discontinuation; TTD2, time to treatment discontinuation sequence.

The median 1L TTD was 21.9 (95% CI, 15.2–25.8) months (Fig. 4A). At 1, 2, and 3 years, 38%, 55%, and 78% of patients, respectively, had discontinued their 1L therapy. During the study period (2017–2023), 209 patients (77%) discontinued their 1L therapy (Fig. 2). While 117 patients (56%) discontinued 1L therapy and switched to a 2L treatment within 2 months of ending 1L therapy, 92 patients (44%) did not receive a 2L therapy. Forty-five patients (22%) died, with a median (95% CI) time to death of 4.0 (2.8–22.9) months after stopping 1L therapy. The remaining patients who discontinued 1L treatment were censored due to switching to a 2L treatment (n = 117, 56% within 2 months; n = 11, 5% after 2 months of ending 1L treatment), ending 1L treatment but remaining in the Flatiron network without treatment (n = 11, 5%), or ending 1L treatment and being lost to follow-up (n = 25, 12%).

### 3.4. Outcomes of subsequent therapies after discontinuation of 1L second-generation ALK TKI

Among 117 patients who received 2L therapy in this study, the median 2L TTD was 7.3 months (95% CI, 5.3–10.2) (Fig. 4B). At 1, 2, and 3 years, 68%, 89%, and 98% of patients, respectively, discontinued 2L therapy. Sensitivity analyses using alternate grace periods, including 30, 90, and 120 days, to quantify 1L and 2L TTD generated highly consistent results (Table 2). When patients were stratified by type of 2L therapy received, TTD on 2L ALK TKI–based therapy (n = 101) was 9.4 months (95% CI, 6.8–12.3) and TTD on 2L non-ALK TKI–based therapies (n = 16) was 4.7 months (95% CI, 3.2–5.2) (Supplementary Figure S1). Ninety-two patients (79%) discontinued their 2L therapy by the data cutoff. The main reason for 2L discontinuation was either a switch or add-on to a third-line (3L) treatment (n = 47, 51%) (Fig. 2). Among 92 patients who discontinued 2L treatment, 45 (49%) did not receive a 3L treatment (25 patients died and 20 ended their 2L treatment episode).

### 3.5. TTD on sequential 1L and 2L therapy (TTD2)

From 2017 to 2023, 184 patients (67%) met the definition of having a TTD2 event, 47 of whom switched to a 3L treatment within 2 months of ending 2L therapy. The remaining 137 patients did not receive a 3L treatment, including 52 patients (38%) who died and 85 patients (62%) who had no evidence of subsequent treatment.

The median TTD2 was 29.4 months (95% CI, 25.1–36.1) for the entire treatment period (Fig. 4C). At 1, 2, and 3 years, 27%, 46%, and 67% of patients, respectively, discontinued their treatment sequence. Median TTD2 in patients who received second-generation ALK TKI followed by third-generation ALK TKI as monotherapy or in combination with another treatment (n = 69, 29.5 [95% CI, 20.5–38.6] months) was longer than in patients who received second-generation ALK TKI followed by other therapies (n = 48, 21.1 [95% CI, 14.4–27.6] months) (Supplementary Figure S2). Sensitivity analyses using alternate grace periods to quantify TTD2 demonstrated consistent results (Table 2). No meaningful differences were observed in 1L TTD or TTD2 when stratified by clinical variables of interest (Supplementary Table S3). Patients treated in academic practices had longer 1L TTD (25.1 vs 18.6 months) and longer TTD2 (38.2 vs 27.0 months) than patients treated in community practices.

### 3.6. Total time on sequential ALK TKI therapy (beyond 2L)

From 2017 to 2023, 185 patients (68%) discontinued sequential ALK TKI therapy. Among those 185 patients, 50 patients (27%) switched to or added on a non–ALK TKI treatment and 135 patients (73%) did not receive additional treatment after discontinuation of sequential ALK TKIs.

The median total time on sequential ALK TKI treatment was 28.0 months (95% CI, 23.6–32.9) (Fig. 4D). At 1, 2, and 3 years, 29%, 48%, and 70% of patients, respectively, discontinued sequential ALK TKI therapy. During the study period, the majority of patients (63%) used one unique ALK TKI, while the remaining patients used 2 (30%) or 3 or more (6%) distinct ALK TKIs. Sensitivity analyses that explored alternate definitions of time on sequential ALK TKI treatment and allowed for the addition of 2 cycles of chemotherapy or combination of ALK TKI with other agents generated consistent findings (Supplementary Table S4).

## 4. Discussion

In this large US-based real-world study of patients with *ALK*-positive advanced NSCLC treated with 1L alectinib or brigatinib, we describe contemporary sequencing patterns and treatment effectiveness of 1L and 2L treatments. During the study period, alectinib was largely the preferred 1L treatment over brigatinib (97% vs 3%). The most common treatment sequence was 1L alectinib followed by 2L lorlatinib. The median 1L TTD of second-generation ALK TKIs was 21.9 months, with 35% discontinuing 1L therapy within 1 year of initiation.

The 1L TTD observed in this real-world study is consistent with the results from the recent real-world study of alectinib (real-world TTD of 23.1 months; median real-world PFS of 24.5 months) [19] and with the results of randomized phase 3 ALEX and ALTA-1L trials, which reported median PFS by BICR of 25.7 months with alectinib and 24.0 months with brigatinib [6,7]. While 1L lorlatinib was outside the scope of this study given the time period, median PFS by BICR and investigator in the 1L setting had not been reached after approximately 3 and 5 years, respectively [9,20]. Median 2L TTD in this real-world study is also concordant with median PFS reported for patients receiving treatment following at least one second-generation ALK TKI (range, 3.8–12.8 months) [11,21-23]. In the present study, treatment duration was longer in the 1L than in the 2L, which confirms the results of other studies and cancer subtypes in which treatment effectiveness declined with each additional line of therapy [24-26]. Patients are therefore most likely to derive the greatest benefit of the most effective ALK TKIs in the 1L setting.

In this study, the median TTD2 was 29.4 months, similar to that in the subgroup of patients receiving sequential 1L second- and then 2L third-generation ALK TKIs (29.5 months). The duration of this sequential therapy appears substantially shorter than the median PFS of 1L lorlatinib in the CROWN trial (not reached after approximately 5 years), [9] although caution must be exercised when comparing findings from this retrospective study with those of a clinical trial. In addition to efficacy, the safety and tolerability of different ALK TKIs should be considered and appropriately monitored and managed based on current recommendations [27,28].

Of note, among those who discontinued 1L therapy in this study, 22% of patients died. The median time to death was 4.0 months after ending 1L therapy without starting 2L therapy. Among the 12% of patients who discontinued 1L therapy and were lost to follow-up, additional patients likely died without further treatment. These findings confirm the high attrition rate seen in other studies in patients with *ALK*-positive advanced NSCLC [7,8,12] and further support the up-front use of the most effective ALK TKI, when tolerated. This approach—rather than that of reserving therapies for later lines, in which the benefit may not be as significant and not all patients remain eligible to receive them—could enable the maximal chance of deriving durable disease control from available therapies for as many patients with *ALK*-positive advanced NSCLC as possible.

This study has several strengths. First, to our knowledge, this is the largest contemporary real-world cohort study of patients with *ALK*-positive advanced NSCLC conducted to date. Second, we provide contemporary data on sequencing patterns to help contextualize treatment decision-making in an era where several next-generation ALK TKIs are available for consideration. Third, this study addresses a clinical gap on sequential outcomes, which was not considered in randomized controlled trials, providing complementary data. Fourth, TTD was used as the primary endpoint for this study and is a pragmatic endpoint for real-world studies, closely associated with PFS [18]. Fifth, results were highly consistent across sensitivity analyses, illustrating the robustness of study assumptions. This includes different grace period lengths, accounting for shorter and longer treatment gaps to account for real-world nonadherence and EHR data collection. Finally, we used a rich and robust source of data that is commonly used to generate real-world evidence in oncology [19,29], providing estimates that are generalizable throughout the US.

As with all observational studies, this study had limitations. Flatiron Health EHR data are collected for administrative purposes; therefore, not all clinical, pathologic, or molecular variables were available (e.g., performance status, sites of metastases including brain involvement, underlying *ALK* fusion variant or co-mutations, mechanisms of drug resistance, and detailed comorbidities). Although TTD is a useful surrogate for PFS [18], TTD may overestimate PFS if patients were to stay on therapy post disease progression as recommended in certain guidelines [4,5,30]. Additionally, data on median overall survival (OS) were not available for this study, which also limits our ability to assess the long-term benefits of different treatment sequences; median OS endpoints have also not yet been reported in the pivotal randomized controlled trials [7-9]. Reasons for treatment discontinuation were not available in the Flatiron Health EHR database, and some patients could have been lost to follow-up if they transitioned to care outside of the Flatiron data network [31]. Given this lack of data, we were unable to determine whether patients discontinued treatment due to adverse events or lack of treatment effectiveness. While this was not an objective of this study, it remains an inherent limitation of Flatiron Health EHR data. We were unable to measure treatment adherence; thus, treatment effectiveness may be overestimated. In addition, the results from this study may not be generalizable to settings outside of the US. As Flatiron mainly contains community practice data, comparison between community and academic practice should not be overinterpreted.

## 5. Conclusions

Overall, we described contemporary sequencing patterns of second- and third-generation ALK TKIs. The sequencing of 1L second-generation ALK TKI followed by 2L third-generation ALK TKI in the real-world setting led to approximately 2.5 years of treatment, but 22% of patients died after stopping 1L therapy without receiving 2L therapy, and over one-third of patients experienced failure of 1L second-generation therapy within 1 year. These findings underscore the importance of considering more effective therapies in the 1L setting—such as the third-generation ALK TKI, lorlatinib, which has demonstrated a 5-year PFS rate of 60%—to improve outcomes in this patient population.[9].

## Supplementary Material

MMC1

## Figures and Tables

**Table 1:** Baseline characteristics of patients initiating 1L therapy with alectinib or brigatinib in the nationwide flatiron health EHR–derived de-identified database.

Characteristic	1L therapy with alectinib or brigatinib (N=273)
**Age, median (Q1, Q3), years**	64 (53, 73)
**Sex, n (%)**	
Female	157 (58)
Male	116 (42)
**Race, n (%)**	
White	175 (64)
Asian	26 (10)
Black or African American	15 (5)
Hispanic or Latino	1 (<1)
Other race	28 (10)
Missing/unknown	28 (10)
**Smoking history, n (%)**	
Yes	124 (45)
No	149 (55)
**Stage at initial diagnosis, n (%)**	
Stage I	20 (7)
Stage II	13 (5)
Stage III	26 (10)
Stage IV	55 (20)
Not documented	159 (58)
**Histology, n (%)**	
Non-squamous cell carcinoma	264 (97)
Squamous cell carcinoma	7 (3)
NSCLC histology NOS	2 (1)
**Practice type, n (%)**	
Academic	81 (30)
Community	184 (67)
Both	8 (3)
**ECOG PS, n (%)**	
0–1	153 (56)
2+	30 (11)
Missing	90 (33)
**ALK test type, n (%)**	
FISH	145 (53)
NGS	89 (33)
IHC	27 (10)
Other, unknown	12 (4)
**ALK sample type, n (%)**	
Tissue	220 (81)
Blood	48 (18)
Unknown	5 (2)
**1L ALK TKI, n (%)**	
Alectinib	264 (97)
Brigatinib	9 (3)

1L, first-line therapy; ALK, anaplastic lymphoma kinase; ECOG PS, Eastern Cooperative Oncology Group performance status; EHR, electronic health record; FISH, fluorescence in situ hybridization; IHC, immunohistochemistry; NGS, next-generation sequencing; NOS, not otherwise specified; NSCLC, non-small cell lung cancer; Q1, first quartile; Q3, third quartile; TKI, tyrosine kinase inhibitor.

**Table 2:** Results of primary and sensitivity analyses of TTD in the 1L, 2L, and sequence.

	60-day grace period^a^	30-day grace period	90-day grace period	120-day grace period
**1L TTD, n**	**273**	**273**	**273**	**273**
TTD, KM median (95% CI), months	21.9 (15.2–25.8)	20.9 (15.2–25.4)	22.8 (15.4–26.1)	22.8 (16.1–26.6)
Time on treatment, n (%)				
1 year	170 (62)	168 (62)	173 (63)	173 (63)
2 years	122 (45)	121 (44)	125 (46)	125 (46)
3 years	61 (22)	59 (22)	61 (22)	62 (23)
**2L TTD, n**	**117**	**108**	**121**	**122**
TTD, KM median (95% CI), months	7.3 (5.3–10.2)	7.1 (5.1–11.2)	8.5 (6.0–11.2)	9.0 (7.1–11.7)
Time on treatment, n (%)				
1 year	37 (32)	33 (31)	40 (33)	40 (33)
2 years	13 (11)	11 (10)	14 (12)	14 (11)
3 years	2 (2)	2 (2)	3 (2)	3 (2)
**TTD2, n**	**273**	**273**	**273**	**273**
TTD2, KM median (95% CI), months	29.4 (25.1–36.1)	28.0 (23.8–32.3)	29.7 (26.4–37.1)	29.6 (26.6–36.6)
Time on treatment, n (%)				
1 year	200 (73)	194 (71)	205 (75)	206 (75)
2 years	148 (54)	143 (52)	153 (56)	153 (56)
3 years	89 (33)	86 (32)	91 (33)	92 (34)

1L, first-line; 2L, second-line; CI, confidence interval; KM, Kaplan-Meier; TTD, time to treatment discontinuation; TTD2, time to treatment discontinuation sequence.

a The 60-day grace period represents the base case.

## Appendix A. Supplementary data

Supplementary data to this article can be found online at https://doi.org/10.1016/j.lungcan.2024.107919.

## Abbreviations:

1L: first-line
2L: second-line
3L: third-line
ALK: anaplastic lymphoma kinase
BICR: blinded independent central review
CI: confidence interval
ECOG PS: Eastern Cooperative Oncology Group performance status
EHR: electronic health record
FISH: fluorescence in situ hybridization
IHC: immunohistochemistry
KM: Kaplan-Meier
NGS: next-generation sequencing
NOS: not otherwise specified
NSCLC: non-small cell lung cancer
PFS: progression-free survival
Q1: first quartile
Q3: third quartile
TKI: tyrosine kinase inhibitor
TTD: time to treatment discontinuation
TTD2: time to treatment discontinuation sequence
