# Supplementary material for: Real-world treatment sequencing and effectiveness of second- and third-generation ALK tyrosine kinase inhibitors for ALK-positive advanced non-small cell lung cancer
Source: Lung Cancer. Author manuscript; Available in PMC 2025 Dec 31. (PMC12754696; doi:10.1016/j.lungcan.2024.107919)
Supplement: MMC1 [file NIHMS2126553-supplement-MMC1.pdf]

Supplementary Material: Real-World Treatment Sequencing and Effectiveness of Second- and Third-Generation ALK Tyrosine Kinase Inhibitors for ALK-Positive Advanced Non-Small Cell Lung Cancer

Table S1. Characteristics of Patients Based on Who Discontinued 1L Therapy Within 12 Months From the Index Date

Characteristic	Discontinued 1L therapy within 12 months (n=96)	Continued 1L therapy beyond 12 months (n=177)
**Age, median (Q1, Q3), years**	64 (55, 74)	64 (53, 73)
**Sex, n (%)**		
Female	57 (59)	100 (56)
Male	39 (41)	77 (44)
**Race, n (%)**		
White	55 (57)	120 (68)
Asian	11 (11)	15 (8)
Black or African American	5 (5)	40 (6)
Hispanic or Latino	1 (1)	0 (0)
Other race	12 (13)	16 (9)
Missing/unknown	12 (13)	16 (9)
**Smoking history, n (%)**		
Yes	54 (56)	70 (40)
No	42 (44)	107 (60)
**Stage at initial diagnosis, n (%)**		
Stage I	3 (3)	17 (10)
Stage II	7 (7)	6 (3)
Stage III	8 (8)	18 (10)
Stage IV	19 (20)	36 (20)
Not documented	59 (61)	100 (56)
**Histology, n (%)**		
Non-squamous cell carcinoma	92 (96)	172 (97)
Squamous cell carcinoma	4 (4)	3 (2)
NSCLC histology NOS	0 (0)	2 (1)
**Practice type, n (%)**		
Academic	27 (28)	54 (31)
Community	65 (68)	119 (67)
Both	4 (4)	4 (2)
**ECOG PS, n (%)**		
0/1	48 (50)	105 (59)
2+	14 (15)	16 (9)
Missing	34 (35)	56 (32)
**ALK test type, n (%)**		
FISH	50 (52)	95 (54)
NGS	32 (33)	57 (32)
IHC	10 (10)	17 (10)
Other, unknown	4 (4)	8 (5)

Characteristic	Discontinued 1L therapy within 12 months (n=96)	Continued 1L therapy beyond 12 months (n=177)
**ALK sample type, n (%)**		
Tissue	79 (82)	141 (80)
Blood	15 (16)	33 (19)
Unknown	2 (2)	3 (2)

1L, first-line; ALK, anaplastic lymphoma kinase; ECOG PS, Eastern Cooperative Oncology Group performance status; FISH, fluorescence in situ hybridization; IHC, immunohistochemistry; NGS, next-generation sequencing; NOS, not otherwise specified; NSCLC, non-small cell lung cancer; Q1, first quartile; Q3, third quartile.

Table S2. Characteristics of Patients Who Did Not Receive 2L Therapy After Discontinuing 1L Therapy

Characteristic	Patients not receiving 2L after discontinuing 1L (n=92)
**Age, median (Q1, Q3), years**	66 (55, 75)
**Sex, n (%)**	
Female	55 (60)
Male	37 (40)
**Race, n (%)**	
White	54 (59)
Asian	11 (12)
Black or African American	4 (4)
Hispanic or Latino	0 (0)
Other race	13 (14)
Missing/unknown	10 (11)
**Smoking history, n (%)**	
Yes	45 (49)
No	47 (51)
**Stage at initial diagnosis, n (%)**	
Stage I	9 (10)
Stage II	2 (2)
Stage III	11 (12)
Stage IV	15 (16)
Not documented	55 (60)
**Histology, n (%)**	
Non-squamous cell carcinoma	91 (99)
Squamous cell carcinoma	1 (1)
NSCLC histology NOS	0 (0)
**Practice type, n (%)**	
Academic	22 (24)
Community	68 (74)
Both	2 (2)
**ECOG PS, n (%)**	
0/1	50 (54)
2+	16 (17)
Missing	26 (28)
**ALK test type, n (%)**	
FISH	50 (54)
NGS	29 (32)
IHC	9 (10)
Other, unknown	4 (4)
**ALK sample type, n (%)**	
Tissue	75 (82)
Blood	16 (17)
Unknown	1 (1)

1L, first-line; 2L, second-line; ECOG PS, Eastern Cooperative Oncology Group performance status;

FISH, fluorescence in situ hybridization; IHC, immunohistochemistry; NGS, next-generation

sequencing; NSCLC, non-small cell lung cancer; NOS, not otherwise specified; Q1, first quartile; Q3, third quartile.

Table S3. 1L TTD and TTD2 Stratified by Key Baseline Characteristics

	ECOG PS			Practice Type			Age		Sex	
	**0/1 (n=153)**	**2+ (n=30)**	**Missing (n=90)**	**Academic (n=81)**	**Community (n=184)**	**Both (n=8)**	**<65 years (n=141)**	**>65 years (n=132)**	**Male (n=116)**	**Female (n=157)**
1L TTD, KM median (95% CI)	24.6 (15.4-32.7)	14.1 (6.8-32.4)	18.3 (13.3-24.6)	25.1 (15.9-35.1)	18.6 (14.2-25.4)	15.6 (3.5-NR)	20.2 (14.1-27.3)	22.8 (14.3-28.5)	25.8 (16.8-32.7)	16.5 (13.7-24.6)
Time on treatment, n (%)										
1 year	101 (66)	15 (50)	54 (60)	51(63)	115 (63)	4 (50)	89 (63)	81 (61)	74 (64)	96 (61)
2 years	74 (48)	12 (40)	36 (40)	37 (46)	81 (44)	4(50)	61 (43)	61 (46)	57 (49)	65 (41)
3 years	40 (26)	7 (23)	14 (16)	24 (30)	35 (19)	2 (25)	32 (23)	29 (22)	23 (20)	38 (24)
TTD2, KM median (95% CI)	33.3 (27.0-44.0)	20.5 (11.5-37.0)	25.8 (18.6-37.6)	38.2 (25.2-46.8)	27.0 (21.4-32.9)	28.1 (7.3-NR)	28.5 (23.4-39.7)	29.7 (23.8-37.6)	33.3 (25.6-44.3)	27.0 (21.4-32.3)
Time on treatment, n (%)										
1 year	119 (78)	18 (60)	63 (70)	61 (75)	133 (72)	6 (75)	106 (75)	94 (71)	85 (73)	115 (73)
2 years	88 (58)	13 (43)	47 (52)	48 (59)	95 (52)	5 (63)	74 (52)	74 (56)	67 (58)	81 (52)
3 years	57 (37)	8 (27)	24 (27)	33 (41)	54 (29)	2 (25)	48 (34)	41 (31)	36 (31)	53 (34)

1L, first-line; CI, confidence interval; ECOG PS, Eastern Cooperative Oncology Group performance status; KM, Kaplan-Meier; NR, not reached; TTD, time to treatment discontinuation; TTD2, time to treatment discontinuation sequence.

Table S4. Sensitivity Analyses Exploring Alternate Definitions of Time on Sequential ALK TKI Treatment

	N=273	
	**Allow continuation on chemotherapy up to 2 cycles or 8 weeks**	**Allow continuation on ALK inhibitor combination with other drugs**
**TTD on sequential ALK TKI, months**		
KM, median (95% CI)	29.5 (24.8-33.5)	29.5 (25.6-36.3)
**Time on ALK TKI, n (%)**		
1 year	203 (74)	199 (73)
2 years	146 (53)	149 (55)
3 years	84 (31)	90 (33)

ALK, anaplastic lymphoma kinase; CI, confidence interval; KM, Kaplan-Meier; TKI, tyrosine kinase inhibitor; TTD, time to treatment discontinuation.

Figure S1. TTD in patients with *ALK*-positive advanced NSCLC stratified by 2L therapy.

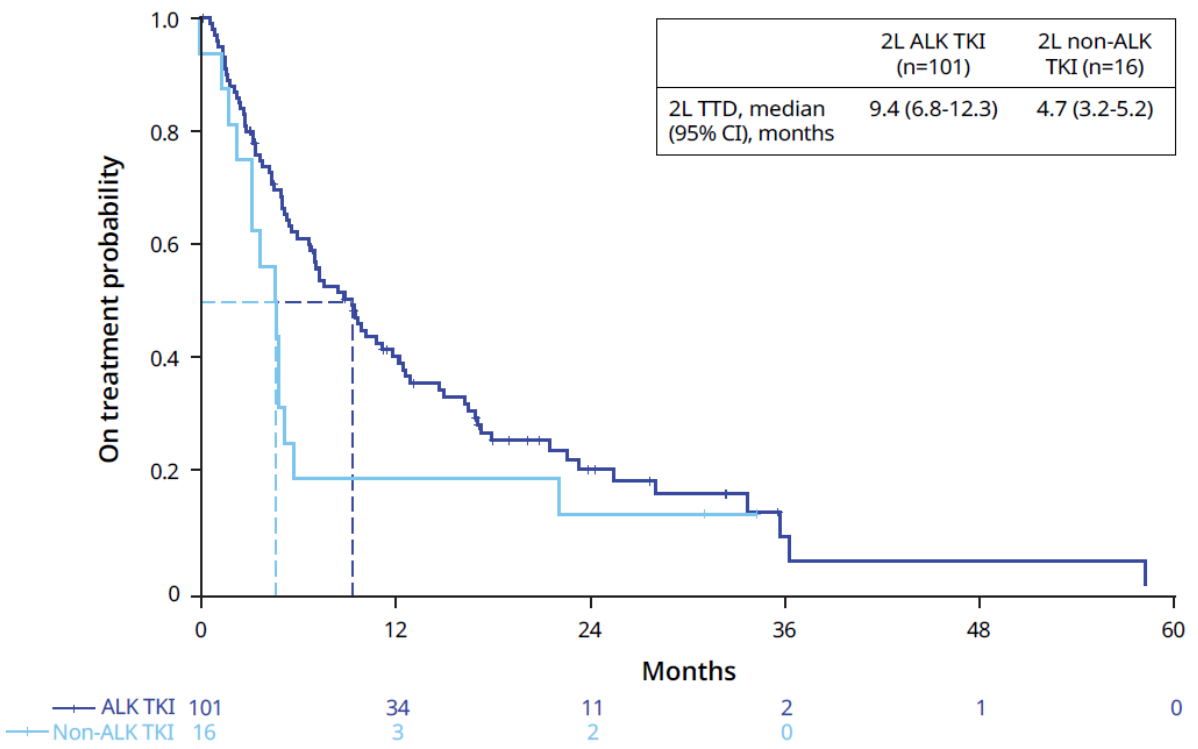

2L, second-line; ALK, anaplastic lymphoma kinase; CI, confidence interval; TKI, tyrosine kinase inhibitor; TTD, time to treatment discontinuation.

Figure S2. Total sequential time on therapy stratified by second-line third-generation ALK TKIs[a] vs all other treatments.

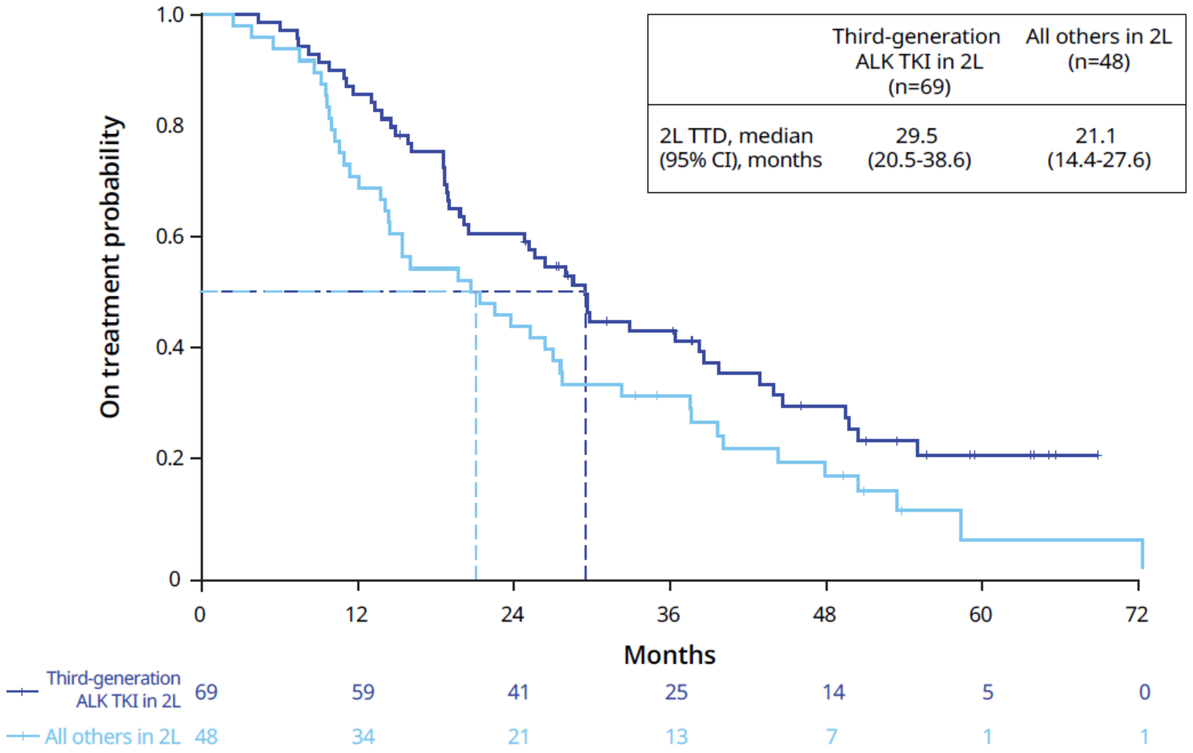

2L, second-line; ALK, anaplastic lymphoma kinase; CI, confidence interval; TKI, tyrosine kinase inhibitor; TTD, time to treatment discontinuation.

[a] Third generation ALK TKI as monotherapy or in combination with another treatment.
